# Development of an Immunodeficient Pig Model for Pancreatic Cancer Xenotransplantation Using Splenectomy, Thymectomy, and Oral Immunosuppression

**DOI:** 10.3390/medicina61040586

**Published:** 2025-03-25

**Authors:** Jun Suh Lee, Yoo-Seok Yoon, Ho-Seong Han, Jai Young Cho, Hae-Won Lee, Boram Lee, Yeshong Park, MeeYoung Kang

**Affiliations:** 1 Department of Surgery, Bucheon Sejong Hospital, Bucheon 14754, Republic of Korea; rudestock@gmail.com; 2 Department of Surgery, Seoul National University Bundang Hospital, Seongnam 13620, Republic of Korea

**Keywords:** animal model, patient-derived xenograft, immunosuppression, pig model

## Abstract

*Background and Objectives*: Animal models are widely used in medical research, but most are limited to small or medium-sized species due to logistical constraints. However, pancreatic cancer research and surgical xenograft models require large animals with anatomical similarities to humans and minimal immune rejection. This study evaluates the feasibility of an operative immunodeficient pig model for patient-derived xenografts. *Materials and Methods*: During the period of October 2020 and October 2021, four pigs were used to establish a pig model at Seoul National University Bundang Hospital. A conventional pig 40 weeks of age was used. After introduction into the animal laboratory, splenectomy and thymectomy were performed to minimize B-cell and T-cell function. One week after the initial operation, oral immunosuppression was administered. After 4 weeks, human PDAC cells were implanted in the liver and pancreas. After 4 weeks of implant, the pigs were sacrificed, and the operative and pathologic findings were analyzed. *Results*: All four pigs survived the 9-week experiment. Indwelling venous catheters for drug-level monitoring were attempted but failed. Splenectomy and thymectomy were deemed to be feasible and effective. Oral immunosuppression was acceptable, but the initial dosage was better tolerated at low levels. Out of the four pigs, one pig showed a mass formation at the cell line injection site, demonstrating reactive cell clusters on pathology. *Conclusions*: This pig model using conventional pigs is a feasible model of immunosuppression. It is necessary to fine-tune the oral immunosuppression dosage and develop methods for the frequent monitoring of immunosuppression levels.

## 1. Introduction

Pancreatic cancer remains one of the most lethal malignancies, with projections indicating that it will become the second leading cause of cancer-related deaths by 2030 [1]. Despite advances in surgical techniques and systemic chemotherapy, overall survival remains dismal, largely due to early micrometastatic disease and a high recurrence rate following resection [2]. Surgical resection is the only treatment option that offers a chance to be cured, but only 15–20% of patients present with resectable disease [1].

Animal models are widely used in the field of medicine. Most animal models are small-animal models, such as rats or mice. These animals have advantages such as rapid breeding capacity and ease of management. However, in fields of research such as cancer therapeutics, large-animal models are important. The proximity of size to humans is a significant advantage since the anatomical and physiological characteristics are closer to humans compared to small or medium-sized animals. Pigs are primarily used for large-animal models. They have a high growth rate with short generation intervals, and their breeding techniques have been standardized, facilitating easier research [3]. Pig models are being utilized widely in the field of surgical technique, genetic diseases, and translational research, to name a few [4].

Patient-derived xenograft (PDX) models are a topic of much interest, with active research being performed using many kinds of animal models. Cancer tissues from humans are harvested and implanted into an animal model. After this procedure, if the graft survives, many kinds of in vivo experiments can be performed. But a major factor concerning this kind of research is graft rejection. In the absence of appropriate immunosuppression, it has been shown that PDX models fail to grow solid tumors [5]. To alleviate graft rejection, immunodeficient models have been developed. To maximize the advantages of the large-animal model and minimize rejection, transgenic “oncopig” models have been developed [6]. The major disadvantage of this model is the difficulty in genetic engineering and the cost. Many, if not most, laboratories do not have the resources to conduct research with this kind of model.

In 2019, Itoh et al. published a study describing their model of an immunodeficient pig model, which is established by performing splenectomy, thymectomy, and oral immunosuppression [7]. This method, known as the “operative immunodeficient pig model (OIDP)” by the authors, uses conventional wild-type pigs. Since this method does not require any genetic engineering, it is much more cost-effective. The establishment of this model using a standardized protocol may enable many more researchers to join this field of research.

However, despite its potential, the OIDP model has not been widely reproduced or optimized, raising concerns about its reproducibility and feasibility. The main question addressed in this study is whether the OIDP model can be successfully replicated using conventional pigs and adapted for PDX studies in pancreatic ductal adenocarcinoma (PDAC). We hypothesized that by standardizing the OIDP protocol and validating it via PDX tumor engraftment, we could establish a cost-effective, reproducible large-animal model for pancreatic cancer research.

Therefore, we attempted to reproduce and optimize the OIDP model using conventional pigs. Thereafter, we performed a PDX using a human pancreatic ductal adenocarcinoma (PDAC) cell line. By confirming the feasibility of this model, our study aims to bridge the gap between small-animal models and clinically relevant large-animal research in pancreatic cancer.

## 2. Materials and Methods

(1)  Animals

In this study, 4 conventional crossbreed pigs were used (Yorkshire, Berkshire, Duroc). All pigs were male and aged between approximately 40 weeks, with a weight of approximately 20 to 25 kg. The animals were housed in a designated animal research facility at Seoul National University Bundang Hospital, where they were monitored daily for signs of infection, distress, or complications for the entire duration of the study (from surgery to sacrifice, lasting approximately 8 weeks). This study was performed in strict accordance with the recommendations in the Guide for the Care and Use of Laboratory Animals of the National Institutes of Health. The experiment protocol was approved by the Institutional Animal Care and Use Committee of Seoul National University Bundang Hospital. (Approval number BA-2010-305-092-01).

(2)  Surgical Technique

**Figure 1:**
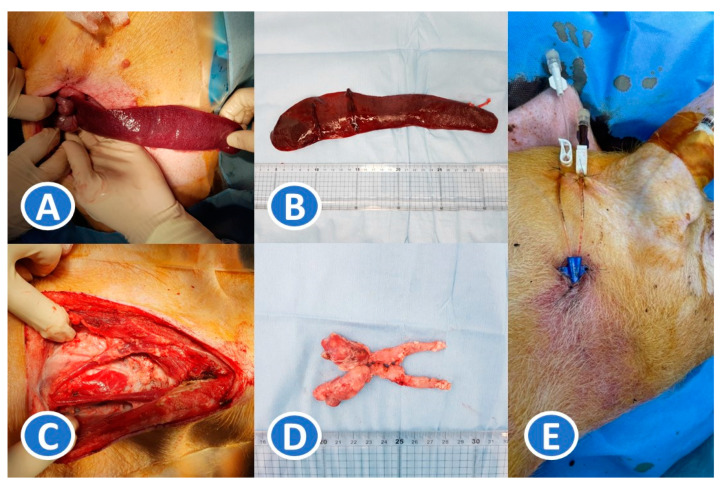
Development of an immunodeficient pig model using splenectomy, thymectomy, and oral immunosuppression. (**A**,**B**): Splenectomy. (**C**,**D**): Thymectomy. (**E**): Insertion of an internal jugular catheter for monitoring immunosuppression levels. The catheter was tunneled through the subcutaneous fat layer and anchored on the posterior neck region.

To facilitate B- and T-cell suppression, thymectomy and splenectomy were performed (Figure 1A–D). All surgical procedures were performed in a sterile operating room within the animal research facility at Seoul National University Bundang Hospital. The pigs were anesthetized with 2.2% sevoflurane inhalation anesthesia (Sevofran^®^, Hana Pharm. Co., Ltd., Seoul, Republic of Korea). A midline incision was made in the neck, and a thymectomy was performed. In one case, a caudal extension of the lower pole of the thymus required thoracic extension of the incision and partial sternal division. After complete removal of the thymus, the internal jugular vein was punctured directly, and a double lumen catheter (Arrow double lumen central venous catheter, 5Fr, Teleflex^®^, Morrisville, NC, USA) was inserted (Figure 1E). The catheter was tunneled through the subcutaneous fat layer and anchored to the posterior neck region to facilitate frequent monitoring of immunosuppression levels. After wound closure, an upper midline incision was made in the abdomen, and a splenectomy was performed. The average duration of each surgery was approximately 3 h, including preparation and postoperative stabilization. The pig was given a recovery period of 1 week. After 1 week, oral immunosuppression started.

(3)  Oral immunosuppression protocol

Oral immunosuppression was initiated 7 days after the surgical procedure to allow for recovery from surgery before the introduction of immunosuppressive agents. The administered dosage was tacrolimus 0.5 mg/kg and mycophenolate mofetil (MMF) 500 mg twice a day. According to a previously published oral immunosuppression protocol for pigs, steroids were not given [6]. The medication was crushed into powder and mixed with feed for ingestion. We attempted to monitor the tacrolimus trough levels to maintain them between 5 and 15 μg/L by performing daily blood testing. Tacrolimus concentration was measured using liquid chromatography–tandem mass spectrometry, a standard method for the precise quantification of immunosuppressive drugs in blood samples. However, as described later in the Results Section, maintaining the venous catheter proved to be difficult, and drug-level monitoring was performed only once weekly. Sampling was performed by inducing general anesthesia using 2.2% sevoflurane inhalation anesthesia (Sevofran^®^, Hana Pharm. Co., Ltd., Seoul, Republic of Korea).

(4)  Patient Derived Xenograft

**Figure 2:**
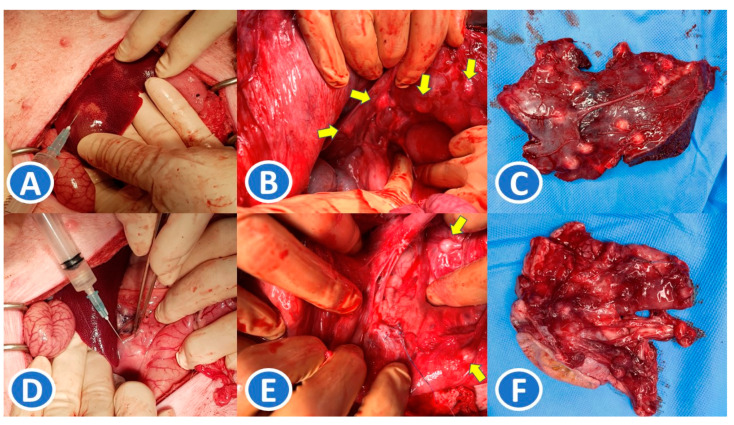
Injection of human pancreatic cancer cell line and operative findings. (**A**,**D**): A human pancreatic cancer cell line was injected into the liver and pancreas. (**B**,**C**,**E**,**F**): Operative findings showed multiple whitish nodular lesions around the injection sites at the liver and pancreas. (yellow arrow).

After 4 weeks of oral immunosuppression, a pancreatic cancer cell line was implanted into the liver and pancreas of the pig. To establish a PDX model, a human pancreatic cancer cell line was used (SNU-324, Korean Cell Line Bank, Seoul, Republic of Korea). This cell line contains distinct genetic mutations and is widely used in pancreatic cancer biology studies [7]. Cell culture was performed, and 4 mL of cell line fluid was prepared to include approximately 3 × 10^9^ cells for implant. The previous upper midline incision was re-opened, and cell line fluid was injected into the liver and pancreas. (Figure 2A,D). The injected areas were marked by sutures for later identification. After implanting the cell line, the pigs were maintained on oral immunosuppression for 4 weeks. After this period, the pigs were sacrificed using an intravenous injection of potassium chloride (JW pharmaceutical, Gwacheon-si, Republic of Korea) under deep anesthesia to ensure humane sacrifice. The liver and pancreas were harvested and sent for pathologic examination.

(5)  Histopathological Assessments

Histological examination was performed on harvested tissues to assess tumor engraftment and tissue response at the implantation sites. Tissues were fixed in 10% neutral-buffered formalin, embedded in paraffin, sectioned, and stained with hematoxylin and eosin (H&E).

**Figure 3:**
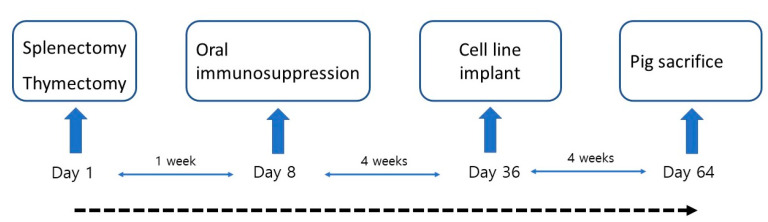
Outline of operative immunodeficient pig model protocol.

To examine the mass lesion, microscopic evaluation focused on identifying tumor cells. In cases where no mass lesions were observed, the implanted areas were examined for residual cell clusters or inflammatory changes. All samples were reviewed by a pathologist to determine whether viable cancer cells were present or if only reactive changes had occurred. The outline of the entire experiment is described in Figure 3.

## 3. Results

All four pigs survived the 9-week experiment. Initially, indwelling venous catheters were planned for frequent drug-level monitoring, but displacement occurred in two animals due to the pigs rubbing the catheters against the wall. In the other two pigs, catheter placement was not attempted, and weekly blood sampling was performed under general anesthesia.

The splenectomy and thymectomy operations were uneventful in most cases. In pig 3, the neck midline incision was insufficient for thymectomy since the gland extended down into the sternum. Therefore, a caudal extension of the incision with partial sternum division was performed. After the operation, the neck wound developed a large seroma. Due to concerns of infection, a reoperation was performed under general anesthesia. After the two sequential operations, this animal developed severe symptoms of diarrhea, lethargy, and poor oral intake. However, the animal was able to survive for the whole length of 9 weeks.

Oral immunosuppression was given at a dosage of tacrolimus 0.5 mg/kg and mycophenolate mofetil (MMF) 500 mg twice a day. The target trough level of tacrolimus was 5~15 ug/L, and MMF levels were not monitored. We found that initial doses of 0.5 mg/kg severely overshot the target levels. Also, the animals displayed severe diarrhea and lethargy. In later experiments, initial doses were given at approximately half and slowly increased to the recommended dosage. This protocol led to easier control of drug dosages and fewer side effects.

**Figure 4:**
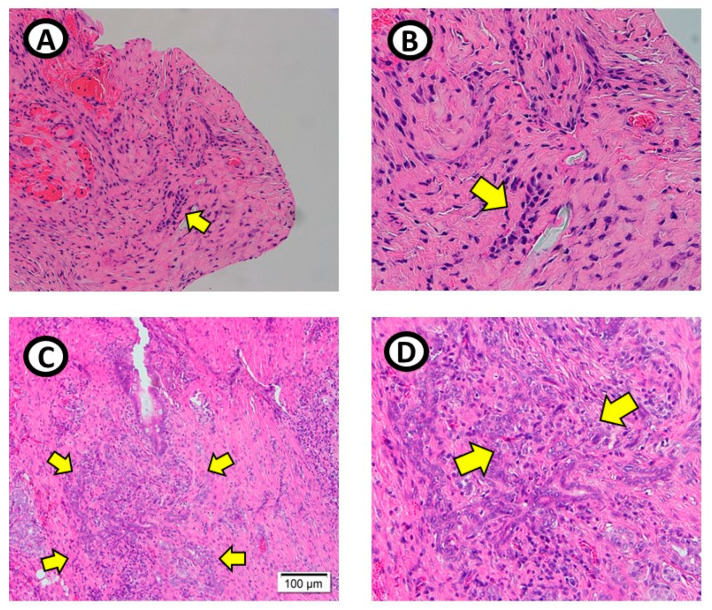
Pathologic findings of the implanted pancreatic cancer cell line. (**A**,**B**): Implanted area of the pancreas with no mass formation. Small areas of reactive cells with inflammation were noted. (yellow arow) (**C**,**D**): Impanted area of the pancreas with multiple nodular masses. Large clusters of reactive cells were identified. On magnification, pleomorphic nuclei were examined. (yellow arrow).

After the pancreatic cancer cell line was implanted, three pigs did not show any operative findings of a mass formation. In one pig, multiple firm and whitish mass lesions were noted at the liver near the hilum and at the pancreatic head near the primary injection site (Figure 2B,C,E,F). Upon pathologic examination, these mass lesions showed inflammation and clusters of reactive cells containing pleomorphic nuclei when magnified (Figure 4C,D). In the other three animals with no mass lesions, pathologic examination of the cell line-implanted area only showed small clusters of reactive cells (Figure 4A,B). No cancer cells were observed. The results of the experiment are described in Table 1.

## 4. Discussion

In this animal study, we aimed to establish a large-animal model of patient-derived xenografts using conventional pig and human PDAC cells. To induce immunosuppression, splenectomy and thymectomy were performed on pigs, and oral immunosuppression was provided. After 4 weeks of oral immunosuppression, human PDAC cells were directly implanted in the pancreas and liver. Four weeks after implantation, the pigs were sacrificed for direct examination, organ harvest, and pathologic examination.

We failed to establish a pancreatic cancer model because cancer cells were absent in the harvested liver or pancreas. Although this was a discouraging finding, we were able to successfully reproduce the operative immunodeficient pig model. We learned that this was a difficult model to establish due to the fragile physical state of the animals caused by surgery and oral immunosuppressive agents.

Pig models are currently being used in many different fields of medicine. The similarity of pigs to humans regarding organ size, anatomy, and physiology enables pig model research that shows reproducible effects in humans. Research that requires complicated surgical procedures such as renal transplantation, pancreatectomy, or thyroidectomy are some fields in which pig models can be most beneficial [3,8,9,10,11]. Another field of study that can benefit from using pig models is pancreatic cancer. Pancreatic cancer ranks as the fourth most common cause of cancer-related deaths, with an increasing trend in occurrence. Accordingly, multiple topics in pancreatic cancer therapy are being actively debated. However, pig models of pancreatic cancer have largely depended on transgenic oncopigs, which are commercially available but very expensive [12]. If this operative immunodeficient model can be further revised and established, it would enable many more researchers to study this important topic.

Although our study did not achieve tumor engraftment, it provides important insights into the challenges of developing a large-animal PDX model for pancreatic cancer. Immunodeficient pigs have the potential to bridge the gap between small-animal xenograft studies and clinical applications by offering a physiologically relevant environment for tumor growth and therapeutic testing [7,13]. However, the need for sustained immunosuppression, the metabolic and physiological stress imposed on the animals, and the variability in tumor take rates remain critical hurdles [14]. Addressing these challenges through improved immunosuppression protocols, optimized tumor implantation techniques, and extended observation periods could enhance the viability of this model for future research.

We would like to note several difficulties during the experiment, which can be areas with room for improvement in future research.

The first primary difficulty in monitoring immunosuppression was the instability of the central venous catheter. During thymectomy, we inserted a vascular catheter into the jugular vein under direct vision, tunneled it subcutaneously, and secured it to the superior aspect of the neck. Although this approach facilitated blood sampling, the animals frequently rubbed against the enclosure, leading to catheter displacement and eventual loss of function. We suggest that future studies explore the use of subcutaneous chemoports, which are commonly used in clinical settings for long-term venous access. These ports could reduce external exposure and minimize the risk of dislodgement. Additionally, the placement of the catheter in a less accessible site, such as the femoral vein, could be considered to prevent mechanical stress from the pig’s natural behavior.

Secondly, we learned that the recommended dosage of 0.5 mg/kg, when given initially, can be too much for the pig. In the earlier cases, we noticed a dramatic overshooting in drug levels, which were detected late because of the difficulty in monitoring. In later cases, we reduced the initial dose to half and slowly increased the dose, which alleviated the fluctuation in drug levels and the symptoms. A stepwise immunosuppressive dosing strategy should be employed from the outset, along with fine-tuning of the dosage to minimize toxicity and adverse effects.

Third, the compounded stress of surgery and immunosuppression was a significant burden on the animals. After administration of immunosuppression, some animals exhibited diarrhea and lethargy. In these cases, immunosuppression was reduced, which led to an improvement in symptoms but drastic fluctuations in drug trough levels. It is worthwhile to note that pig 3, on which two operations were performed because of a neck seroma, exhibited the most severe symptoms. It seems that additional general anesthesia, even when performed for a short time, can be hazardous to the pig’s condition.

Fourth, the characteristics of the human PDAC cell line that was used may have been inappropriate for this model. Pancreatic cancer has an affinity for metastasis, in many cases forming only small primary masses, even with extensive distant spread of the disease. This characteristic may have been an obstacle to the formation of a mass. Also, the appropriate dosage of the cell line required for injection was not known. It may be helpful to explore other types of cancer cell lines for a solid tumor PDX model.

Finally, a possible reason for the absence of viable tumor cells in our study is that the four-week post-implantation period may have been insufficient for mass formation. Pancreatic cancer is known for its slow tumor progression and often forms only small primary masses, even when extensive metastasis occurs. A longer observation period of six to eight weeks post-implantation could provide sufficient time for tumor development. Additionally, serial imaging studies, such as ultrasound or MRI, could help monitor early tumor progression and inform researchers about the optimal timing for sacrifice.

This study has several limitations that should be acknowledged. First, no imaging follow-up was conducted to monitor tumor engraftment. While the animals were sacrificed at a predetermined time point, the absence of serial imaging, such as ultrasound or MRI, limited our ability to assess tumor progression over time. Second, a control group was not included in this study, preventing direct comparison between immunosuppressed and non-immunosuppressed animals. This limitation makes it difficult to differentiate between the effects of immunosuppression and other potential contributing factors, such as surgical stress. Third, we did not perform peripheral blood analysis beyond monitoring tacrolimus levels. Therefore, we could not assess immune cell changes, such as variations in neutrophil, lymphocyte, or monocyte counts, which could have provided additional insights into the degree of immunosuppression. Lastly, the lack of a control group also affects the interpretation of clinical symptoms such as diarrhea, lethargy, and poor oral intake. Although these symptoms are commonly associated with immunosuppressive therapy, we cannot completely rule out other causes without a comparison group. Future studies should incorporate imaging follow-up, comprehensive immune profiling, and appropriate control groups to further refine and validate the operative immunodeficient pig model.

## 5. Conclusions

The operative immunodeficient pig model is a feasible large-animal xenograft model, but challenges remain in immunosuppression stability, surgical recovery, and tumor engraftment. This study highlights the limitations of oral immunosuppression and the need for refined monitoring strategies. Our findings contribute to the development of cost-effective large-animal cancer models by identifying key areas for improvement.

Future studies should focus on optimizing immunosuppression protocols, incorporating noninvasive tumor monitoring, extending engraftment duration, and including a control group to better assess treatment effects. With further refinement, ths model could provide a more accessible alternative to transgenic pig models for pancreatic cancer research and therapeutic testing.

## Figures and Tables

**Table 1:** Complications, operative findings, and pathologic findings of the operative immunodeficient pig model.

	Pig 1	Pig 2	Pig 3	Pig 4
method of drug-level monitoring	central venous catheter	central venous catheter	weekly sampling	weekly sampling
splenectomy and thymectomy complications	none	none	wound seroma at neck incision	none
immunosuppression complications	diarrhea and lethargy	diarrhea and lethargy	diarrhea, lethargy, and poor oral intake	diarrhea
operative findings	no mass formation	no mass formation	no mass formation	mass formation at the liver and pancreas
pathologic findings	no findings	small clusters of reactive cells	small clusters of reactive cells	mass formation at the liver and pancreas

## Data Availability

The original contributions presented in this study are included in the article. Further inquiries can be directed to the first author (J.S.L., rudestock@gmail.com).
